# Diverse Landscape of Tunable Magnetic, Topological, and Ferroelectric States in 2D Ti_3_Se_3_Te_2_

**DOI:** 10.1002/advs.202524385

**Published:** 2026-04-09

**Authors:** Jiangtao Yu, Jingbo Bai, Yali Yang, Shifeng Qian, Xiaotian Wang, Zhuhong Liu

**Affiliations:** ^1^ Department of Physics University of Science and Technology Beijing Beijing China; ^2^ Institute for Superconducting and Electronic Materials Faculty of Engineering and Information Sciences University of Wollongong Wollongong Australia; ^3^ Anhui Province Key Laboratory for Control and Applications of Optoelectronic Information Materials Department of Physics Anhui Normal University Wuhu Anhui China

**Keywords:** altermagnetism, ferroelectricity, quantum anomalous Hall effect, quantum spin Hall effect

## Abstract

2D layered materials provide a powerful platform for exploring the intertwined physics of magnetism, topology, and ferroelectricity. Here, using first‐principles calculations, we reveal a rich landscape of tunable quantum phases and ferroelectric states in the 2D van der Waals material Ti_3_Se_3_Te_2_, controlled by magnetization orientation, stacking configuration, and interlayer sliding. For the monolayer, Ti_3_Se_3_Te_2_ is identified as a dynamically stable ferromagnet whose magnetization direction drives a phase transition between a trivial metal and a quantum anomalous Hall insulator with a nonzero Chern number. In bilayers, two distinct stacking configurations lead to markedly different behaviors: (i) the AA‐stacking bilayer stabilizes an altermagnetic ordering and hosts a quantum spin Hall insulating phase characterized by a nonzero spin Chern number; and (ii) the AA'‐stacking bilayer exhibits a three‐state in‐plane ferroelectricity, beyond the two‐state out‐of‐plane ferroelectricity reported in many altermagnetic systems. Sliding‐induced switching in this configuration reversibly modulates the in‐plane polarization, the easy‐magnetization axis, and the spin splitting. These results demonstrate that Ti_3_Se_3_Te_2_ integrates tunable topological phases, altermagnetism, and sliding‐induced three‐state in‐plane ferroelectricity, establishing it as a versatile van der Waals platform for low‐energy spintronic technologies, topological quantum science, and next‐generation multifunctional applications.

## Graphical abstract

Ti_3_Se_3_Te_2_ emerges as a multifunctional 2D van der Waals platform. The monolayer is a dynamically stable ferromagnetic quantum anomalous Hall insulator. In bilayers, two stacking configurations yield distinct phases: AA‐stacking hosts an altermagnetic quantum spin Hall insulator, while AA′‐stacking exhibits three‐state in‐plane ferroelectricity switchable via interlayer sliding, enabling reversible control of polarization, the easy‐magnetization axis, and spin splitting.

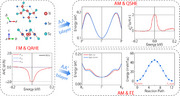

## 1 Introduction

2D multifunctional materials exhibiting magnetic, ferroelectric, and topological characteristics have attracted increasing interest for multifunctional applications [1, 2, 3, 4, 5, 6, 7]. However, most currently reported 2D systems typically exhibit only one or two of these functionalities, and identifying systems with richer functionalities and more exotic physical phenomena remains a major challenge. Developing such advanced 2D multifunctional materials is therefore critically important [8, 9]. To address this challenge, it is therefore highly desirable to develop 2D multifunctional materials that support a diverse and tunable interplay among magnetic, topological, and ferroelectric orders.

Recent progress in 2D materials research has highlighted three promising directions: (i) altermagnetism, a novel magnetic phase. It substantially expands the symmetry and functionality scope of 2D magnets [10, 11, 12, 13, 14]. Unlike ferromagnets (FMs), which suffer from stray fields, or conventional collinear antiferromagnets (AFMs), which typically exhibit spin‐degenerate bands and thus limit spin‐selective transport, altermagnets (AMs) exhibit momentum‐dependent spin splitting while maintaining zero net magnetization. This arises from magnetic sublattices with opposite spins connected by rotational symmetry rather than simple translation or inversion, producing nonrelativistic spin splitting without requiring strong spin‐orbit coupling (SOC) [15, 16, 17, 18]. Consequently, AMs combine the stray‐field resilience of AFMs with the spin‐dependent transport advantages of FM materials, enabling tunable magnetic spin textures via external perturbations such as strain or electric fields and making them a powerful platform for symmetry‐governed spintronics [1, 19, 20, 21, 22, 23]; (ii) magnetic topological phases, particularly quantum spin Hall (QSH) states in 2D AMs. They offer a unique handle for phase realization through symmetry manipulation [24, 25, 26, 27, 28]. Recent works have demonstrated that QSH insulators can emerge in AMs through different mechanisms: either via stacking‐engineered bilayer Chern insulators with compensated magnetization, i.e., bilayer RuCS_3_ [24], or through intrinsic *d*‑wave altermagnetic ordering combined with spin‐orbit and mirror‐spin coupling (e.g., monolayer Fe_2_Te_2_O [25] and multilayer Fe_2_Se_2_O [26]). These developments underscore the importance of identifying new 2D topo‐spintronic systems capable of hosting emerging magnetic topological phases; (iii) a new scheme of sliding ferroelectricity. The sliding ferroelectricity in 2D van der Waals bilayers offers a powerful means to reversibly control polarization and electronic states via breaking inversion symmetry or altering stacking order [1, 3, 20, 29, 30, 31]. When combined with altermagnetism, sliding can simultaneously modulate ferroelectric polarization and momentum‐dependent spin splitting, enabling switchable anomalous Hall signals or sign reversal in magneto‐optical Kerr responses, establishing a promising link between sliding ferroelectricity and spintronic functionalities [1, 19, 20, 32]. However, most reported sliding ferroelectrics are limited to out‐of‐plane polarization with two ferroelectric states [1, 19, 20, 32]. Realizing in‐plane sliding‐induced ferroelectricity with three ferroelectric states coupled to altermagnetism remains rare [33], yet it could provide a powerful way to control spin and polarization, essential for a diverse and tunable state landscape.

Here, using first‐principles calculations, we demonstrate that the 2D van der Waals material Ti_3_Se_3_Te_2_ exhibits widely tunable magnetic, topological, and ferroelectric states through magnetization orientation, stacking order, and interlayer sliding. In the monolayer, it is a dynamically stable ferromagnet, where magnetization direction drives a phase transition between a trivial metal and a quantum anomalous Hall (QAH) insulator. In bilayers, the AA stacking stabilizes an altermagnetic order that hosts a QSH insulating phase, while the AA′ stacking exhibits three‐state in‐plane sliding ferroelectricity, where interlayer sliding reversibly modulates the in‐plane polarization, the easy‐magnetization axis, and spin splitting. These findings establish Ti_3_Se_3_Te_2_ as a unique material unifying topology, altermagnetism, and three‐state in‐plane ferroelectricity, offering new opportunities for next‐generation spintronic and quantum devices.

## 2 Results and Discussion

### 2.1 Tunable Topological Transition in Ti_3_Se_3_Te_2_ Monolayer

**FIGURE 1:**
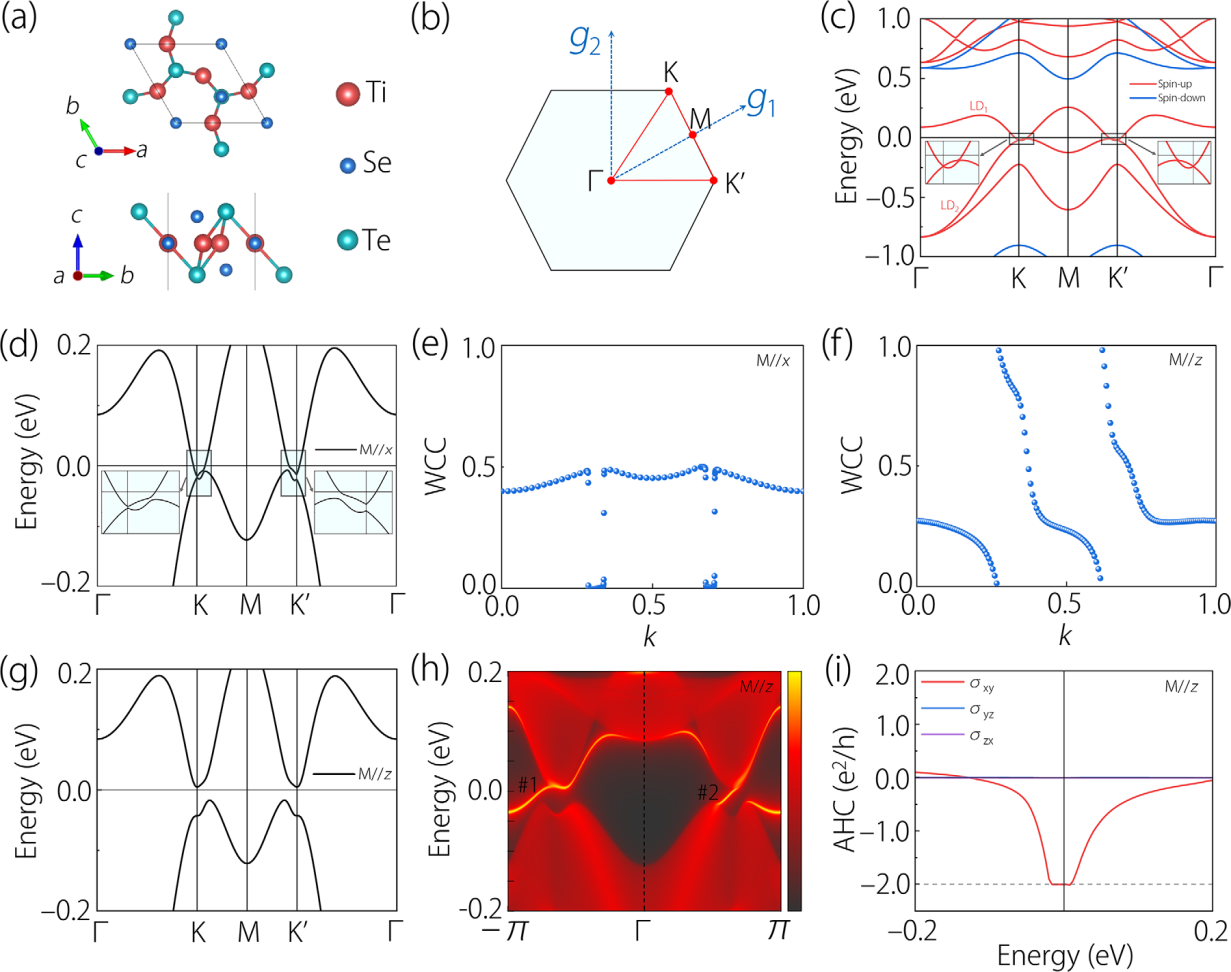
(a) The top and side views of the Ti_3_Se_3_Te_2_ monolayer. The red, blue, and cyan spheres represent Ti, Se, and Te atoms, respectively. (b) Corresponding first Brillouin zone and high‐symmetry paths of band structure calculation. (c) The band structure without SOC. The red and blue curves express the spin‐up and spin‐down channels, respectively. Insets show magnified views of the Weyl point regions. (d) The band structure with SOC, and (e) the evolution of Wannier charge center (WCC) of Ti_3_Se_3_Te_2_ monolayer when the magnetization orientation is aligned along the *x*‐axis. (f) The evolution of WCC when the magnetization is aligned along the *z*‐axis. (g) The band structure with SOC and (h) the edge state of Ti_3_Se_3_Te_2_ monolayer when the magnetization orientation is aligned along the *z*‐axis. (i) The anomalous Hall conductivity (AHC) when the magnetization is aligned along the *z*‐axis.

The Ti_3_Se_3_Te_2_ monolayer crystallizes in a hexagonal lattice with space group *P321* (No. 150), featuring a three‐fold rotation symmetry *C_3z_* and two‐fold rotation symmetry *C_2x_*, as illustrated in Figure 1a. The optimized lattice constants are *a* = *b* = 6.35 Å. Among all considered magnetic configurations, the FM state has the lowest energy configuration, making it more stable than the two AFM configurations and the nonmagnetic (NM) state (Figure S1). The FM primarily originates from the Ti atoms, each carrying a magnetic moment of 0.68 μ_*B*_. The magnetic exchange couplings (J) are summarized in Figure S2a. The positive J value further indicates the FM state is the ground state, consistent with the results for the considered magnetic configurations. The temperature‐dependent magnetization *M*(*T*) curve was obtained through Monte Carlo simulations, and the Curie temperature (*T_C_*) was determined from the peak of the first derivative of the *M*(*T*) curve for different system sizes (see Figure S2b). To assess the reliability of the Monte Carlo simulated *T_C_* and to address potential overestimates due to long‐wavelength fluctuations in the 2D system, we performed systematic finite‐size scaling calculations (see figure S2c). The resulting *T_C_* of 160 K indicates that the Ti_3_Se_3_Te_2_ monolayer retains magnetic order at experimentally accessible temperatures. The formation energy (*E_f_*) is calculated based on the following equation,

(1) $$E_{f}=E\left(Ti_{3}Se_{3}Te_{2}\right)-3E\left(\mathrm{Ti}\right)-3E\left(\mathrm{Se}\right)-2E\left(\mathrm{Te}\right)$$

where *E*(Ti_3_Se_3_Te_2_), *E*(Ti), *E*(Se), and *E*(Te) are the energies of the Ti_3_Se_3_Te_2_ monolayer, the single Ti, Se, and Te atom is their bulk crystal, respectively. The formation energy is −6.90 eV per formula unit (see Table S1), which indicates the chemical stability of the Ti_3_Se_3_Te_2_ monolayer. Phonon spectrum calculations confirm the dynamical stability of the monolayer (Figure S3a). To further validate the stability, *ab initio molecular dynamics* (AIMD) simulations were performed, confirming the thermal stability of the Ti_3_Se_3_Te_2_ monolayer (Figure S3b).

The spin‐resolved band structure of Ti_3_Se_3_Te_2_ monolayer without SOC, together with the corresponding first Brillouin zone are presented in Figure 1c and Figure 1b, respectively. The system exhibits a half‐metallic behavior: the spin‐up bands cross the Fermi level, while the spin‐down channel possesses a large bandgap of 1.4 eV. The *C*_2_ symmetry along the high‐symmetry line protects the crossing of two bands characterized by different *C*_2_ eigenvalues, leading to the formation of Weyl points near the Fermi level. Upon considering the SOC effect, the magnetic moment oriented along the *x*‐direction breaks the *C*_2_ symmetry along the K‐M path, thereby lifting the degeneracy of the Weyl points (see Figure 1d). Wilson loop calculations further reveal a trivial Chern number *C* = 0, as shown in Figure 1e. In contrast, when the magnetization is aligned along the *z*‐axis, the breaking of *C*_2_ symmetry opens a global bandgap of 21.6 meV and triggers the transition into an insulating phase (see Figure 1g). As shown in Figure 1h, two edge states are obvious, indicating a nontrivial topological character. The nontrivial topology is further verified by the evolution of the Wannier charge center (WCC) illustrated in Figure 1f. Therefore, the Ti_3_Se_3_Te_2_ monolayer is a QAH insulator with a Chern number *C* = −2, and the quantized integer Chern number directly signifies the emergence of the QAH effect. In this topological phase, the anomalous Hall conductivity (AHC) is expressed as σ_*xy*_ =  *C* × *e* ^2^/*h*, which describes the quantized Hall plateau at *e* ^2^/*h* and further confirms the quantized nature of transverse transport. We have performed the AHC calculation (see the methods part for details) and the results are shown in Figure 1i. Obviously, a quantized plateau of σ_*xy*_ =   − 2 × *e* ^2^/*h* is observed near the Fermi level, providing clear evidence of the QAH effect in this system.

Note that the calculated magnetic anisotropy energy (MAE) indicates that the in‐plane magnetization is slightly more favorable, with the out‐of‐plane magnetization being 0.37 meV higher in energy (see Figure S2d). This value falls within the typical range of MAE reported for 2D magnetic materials, such as CrBr_3_ (0.16 meV), MnAs (0.28 meV), and CrI_3_ (0.80 meV) (see Figure S4 for a comparison) [34, 35, 36, 37, 38, 39, 40]. Furthermore, in 2D magnetic materials, MAE values in the sub‐meV range are commonly reported and considered sufficient to stabilize long‐range magnetic order against thermal fluctuations. This anisotropy suggests that the magnetization direction can be effectively manipulated, enabling a transition between a metal and a QAH state.

### 2.2 Altermagnetism and Quantum Spin Hall Effect in AA‐Stacking Ti_3_Se_3_Te_2_ Bilayer

**FIGURE 2:**
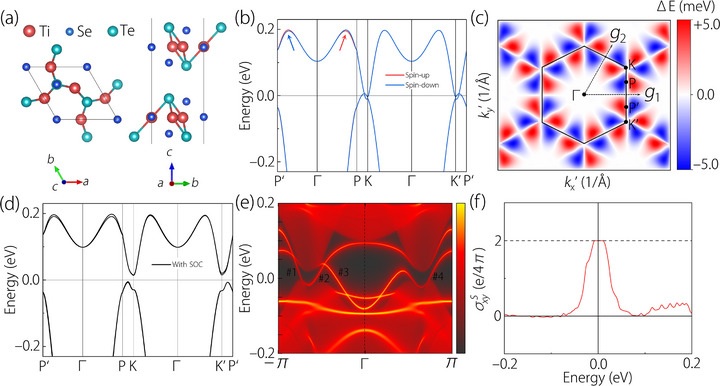
(a) The top and side views of AA‐stacking Ti_3_Se_3_Te_2_ bilayer. The red, blue, and cyan spheres represent Ti, Se, and Te atoms, respectively. (b) The band structure without SOC. The red and blue curves express the spin‐up and spin‐down channels, respectively. (c) The spin splitting in the first Brillouin zone. The red and blue areas denote positive and negative spin splitting of the conduction band, respectively, while the white indicates regions of spin degeneracy. (d) The band structure with SOC, (e) the edge state, and (f) the spin Hall conductivity $\sigma_{xy}^{S}$ when the magnetization is aligned along the *z*‐axis.

Stacking engineering in 2D materials is an effective route to tailor their magnetic and topological states. Figure 2a shows the AA‐stacking Ti_3_Se_3_Te_2_ bilayer, generated by applying a stacking operator $\hat{P}=E$, where *E* is the identity operator, to the monolayer lattice, while preserving the *P321* (No. 150) symmetry. A comparison of the interlayer‐FM and interlayer‐AFM configurations (Figure S5b,c) reveals that the interlayer‐AFM configuration is the ground state. Within the general stacking theory (GST) [21], applying the stacking operator $\hat{P}=E$ to a monolayer introduces an additional in‐plane *C*_2_ symmetry into the bilayers, giving rise to altermagnetism. The spin‐resolved band structure of the Ti_3_Se_3_Te_2_ bilayer without SOC shown in Figure 2b exhibits prototypical altermagnetic features, exhibiting pronounced momentum‐dependent spin splitting along P'–Γ–P high‐symmetry paths. To further quantify the spin splitting in the first Brillouin zone, the energy difference between the conduction band spin‐split states is plotted in Figure 2c, revealing a *i*‐wave symmetry. Red and blue regions denote positive and negative values, respectively, corresponding to the spin‐up band lying above or below the spin‐down counterpart.

To validate the topological and transport effect in the Ti_3_Se_3_Te_2_ bilayer, Figure 2d shows the band structure including SOC with the magnetization aligned along the *z*‐axis. The band crossing points observed without SOC open a gap with a finite value of 18.9 meV, indicating the insulating nature. Figure 2e displays the edge states of the Ti_3_Se_3_Te_2_ bilayer, which exhibit two pairs of helical edge states connecting the valence and conduction bands, confirming the nontrivial topological character. The individual Chern numbers for the two spin manifolds are *C*_+_ =   + 2 and *C*_−_ =   − 2, yielding a total Chern number *C* = 0 and a spin Chern number of *C_S_* = (*C*_+_ − *C*_−_) /2  =   + 2. These results indicate that the system is likely a QSH insulator. Accordingly, the calculated spin Hall conductivity $\sigma_{xy}^{S}=C_{S}\;e/4\pi$ exhibits a quantized plateau within the SOC‐induced gap, as shown in Figure 2f, in good agreement with the spin Chern number *C_S_* =   + 2.

The calculated MAE indicates that the in‐plane magnetization is energetically favorable, with the out‐of‐plane magnetization being only 1.07 meV higher in energy (Figure S5d). Such a weak anisotropy implies that the magnetization reorientation can be effectively switched to the out‐of‐plane (*z*‐axis) direction, allowing precise control over the emergence of a QSH state. Furthermore, to examine the impact of the substrate, a 2 × 2 supercell of the bilayer was matched with a 5 × 5 supercell of h‐BN substrate, resulting in a mismatch of less than 2%. The results show that the altermagnetic state remains the magnetic ground state, with an energy 13.9 meV lower than the FM state (see Table S2), indicating that the substrate acts only as a minor perturbation.

To gain deeper insight into how stacking influences the tunability of the topological phase, we computed the relative energetics of different stacking registries stabilized in the interlayer‐AFM configuration (see Figure S6a). Another stacking configuration with a global energy minimum, generated by using the stacking operator of $\hat{P}=\left\{E|\frac{2}{3},\frac{1}{3}\right\}$ is shown in Figure S6b. Its band structure also displays a momentum‐dependent spin splitting along the high‐symmetry path of P'–Γ–P, while maintaining the *i*‐wave symmetric altermagnetic pattern, as displayed in Figure S6c,d. However, in this case, the system is a trivial insulator rather than a QSH insulator.

### 2.3 Altermagnetism and Sliding‐Induced Three‐State In‐Plane Ferroelectricity in AA' Stacking Ti_3_Se_3_Te_2_ Bilayer

**FIGURE 3:**
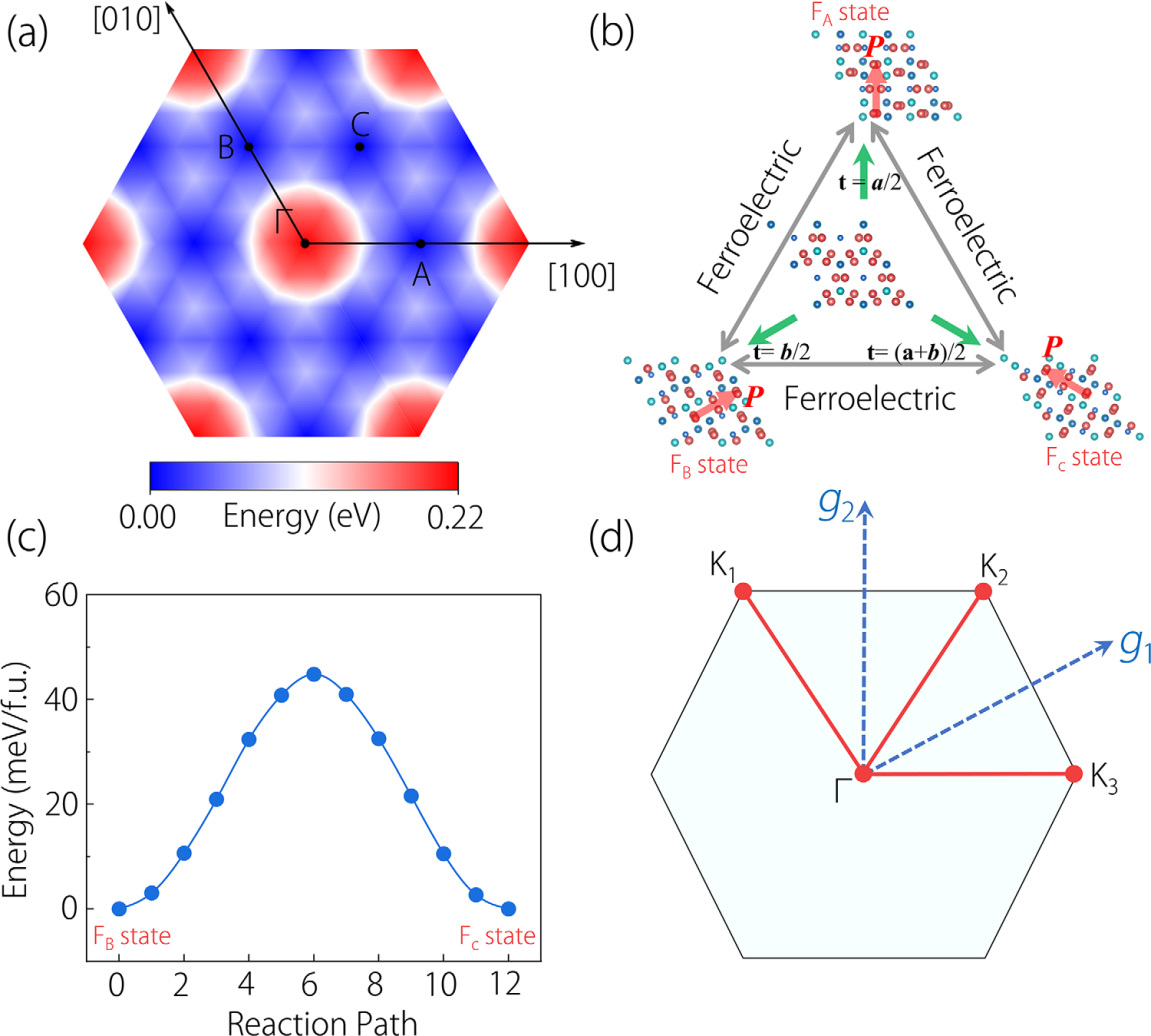
(a) The interlayer‐AFM energy for different translations. (b) Schematic illustration of in‐plane ferroelectric three‐state switching. The red arrows denote the electric polarization direction. (c) Transition energy barriers in the ferroelectric switching path from the *F*_A_ state to the *F*_B_ state. (d) The 2D Brillouin zone of the Ti_3_Se_3_Te_2_ bilayer of three ferroelectric states.

In addition to the AA stacking bilayer, the AA' stacking bilayer, which is generated by applying the stacking operator $\hat{P}=\left\{2_{001}\right\}$, also gives rise to a rich physical landscape. The structure of the AA' stacking configuration, which belongs to the space group *P312* (#149), is displayed in Figure S7a. Ground state calculations demonstrate that the interlayer‐AFM ordering is the most stable magnetic configuration (see Table S3 for details). This stacking also exhibits altermagnetic feature, manifested as a momentum‐dependent spin splitting along the high‐symmetry paths P'–Γ–P, as shown in Figure S7b. According to the general theory of stacking‐induced ferroelectricity in bilayer systems [3], the Ti_3_Se_3_Te_2_ bilayer exhibits three degenerate phases with distinct in‐plane electric polarization, which are related by the *C*_3_ rotational symmetry. Three such phases are generated by the stacking operators $\hat{P}=\left\{2_{001}|\frac{1}{2},0\right\}$, $\hat{P}=\left\{2_{001}|0,\frac{1}{2}\right\}$, and $\hat{P}=\left\{2_{001}|\frac{1}{2},\frac{1}{2}\right\}$, respectively. For clarity, the configurations in which the top layer is shifted by half a lattice constant along the [100], [010], and [110] directions are denoted as the *F*_A_, *F*_B_, and *F*_C_ states. Density functional theory (DFT) calculations were performed to determine the relative energies and the corresponding polarization values of these stacking configurations. As illustrated in Figure 3a, the simply stacked bilayer generated by $\hat{P}=\left\{2_{001}\right\}$ corresponds to the energy maximum. In contrast, sliding the top layer by $\pm \frac{1}{2}$ along the [100], [010], and [110] directions yields sixfold degenerate global energy minima, which are lower than the maximum energy value by 0.22 eV per unit cell, highlighting the pronounced structural stability of these sliding configurations. Ground state calculations further demonstrate that the interlayer‐AFM configuration is the most stable magnetic ordering for all three ferroelectric states (Table S4).

Using the Berry phase method [41], the spontaneous in‐plane ferroelectric polarization of the three degenerate states is evaluated to be 0.31 pC/m after considering the polarization quantum. The polarization vectors point along the [120], [210], and [−110] directions for *F*_A_, *F*_B_, and *F*_C_ states, respectively. The polarization quantum of the Ti_3_Se_3_Te_2_ bilayer is given by $P_{q,i}=\frac{eR_{i}}{\Omega }$, where Ω denotes the in‐plane area of the unit cell and *R_i_* denotes the lattice constant along the polarization direction. This polarization magnitude exceeds that reported for several 2D systems, such as 1T'‐ReS_2_ (0.07 pC/m) and Hf_2_VC_2_F_2_ (0.29 pC/m) [42, 43]. Considering that electric polarization has been experimentally detected in 2D 1T'‐ReS_2_ [42], it should be equally feasible to measure the polarization of the Ti_3_Se_3_Te_2_ bilayer. A schematic illustration of the three‐state in‐plane ferroelectric switching is presented in Figure 3b, where the red arrows indicate the polarization direction. To examine the robustness and reversibility of the ferroelectric states, we mapped the minimum‐energy transition pathways using the climbing‐image nudged elastic band (CI‐NEB) method [44, 45]. As a representative example, the switching process between the *F*_B_ and *F*_C_ configurations is analyzed, involving an interlayer sliding along the [100] direction. The energy barrier for the interlayer sliding between these two states is calculated to be 44.85 meV per formula unit (f.u.) (Figure 3c). This value is higher than that of the CrI_3_ bilayer (23 meV/f.u.) [46], and lower than those reported for GeS bilayer (156meV/f.u.) and GeSe bilayer (66 meV/f.u.) [47], indicating a structural rigidity that suppresses spontaneous sliding while still allowing transitions to be activated by a moderate external electric field.

**FIGURE 4:**
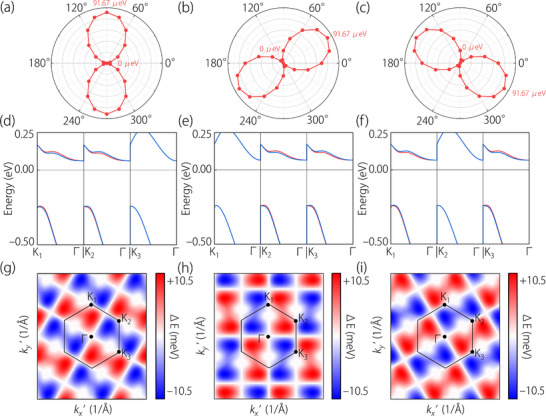
The magnetic anisotropy energy (MAE) of (a) *F*_A_, (b) *F*_B_, and (c) *F*_C_ states. The energy of spin direction along the easy‐magnetization axis is taken as the reference. The spin‐polarized band structure of (d) *F*_A_, (e) *F*_B_, and (f) *F*_C_ states. The spin splitting in the first Brillouin zone of (g) *F*_A_, (h) *F*_B_, and (i) *F*_C_ states. The red and blue areas denote positive and negative spin splitting of the conduction band, respectively, while the white indicates regions of spin degeneracy.

Beyond the polarization switching among the three ferroelectric states, the magnetic orientation and spin‐splitting patterns also undergo significant changes. Due to SOC, the magnetization directions of the three ferroelectric phases are also related by *C*_3_ symmetry. The in‐plane MAE, shown in Figure 4a–c with the lowest total energy set to zero, displays a clear dependence on the magnetization orientation, highlighting a strong interplay between the magnetization direction and the ferroelectric configuration. For all three ferroelectric states, the energy difference between the in‐plane easy and hard magnetization directions is merely 91.67 µeV, indicating extremely weak in‐plane magnetic anisotropy. Interestingly, the easy axes of magnetization align along [100], [010], and [110] for the *F*_A_, *F*_B_, and *F*_C_ states, respectively, revealing a pronounced coupling between the easy magnetization direction and the ferroelectric state. This suggests that the easy‐magnetization axis can be electrically controlled through ferroelectric switching.

Although all three states (*F*_A_, *F*_B_, and *F*_C_) are altermagnetic, their spin splitting exhibits a strong momentum dependence, as reflected in the characteristic band dispersions along the K_1_–Γ–K_2_, K_2_–Γ–K_3_, and K_3_–Γ–K_1_ paths, respectively (Figure 4d–f). To quantify the spin splitting across the first Brillouin zone, the energy difference between the spin‐split conduction bands is plotted in Figure 4g–i. Red (blue) regions mark positive (negative) energy differences, identifying whether the spin‐up conduction band lies above or below the spin‐down one. The spin splitting across the first Brillouin zone is likewise strongly coupled to the ferroelectric states, similar to the behavior of the easy‐magnetization directions. This pronounced coupling suggests that the nonrelativistic spin splitting can be effectively tuned through ferroelectric switching, offering a potential route for electric‐field control of spin‐dependent properties.

**FIGURE 5:**
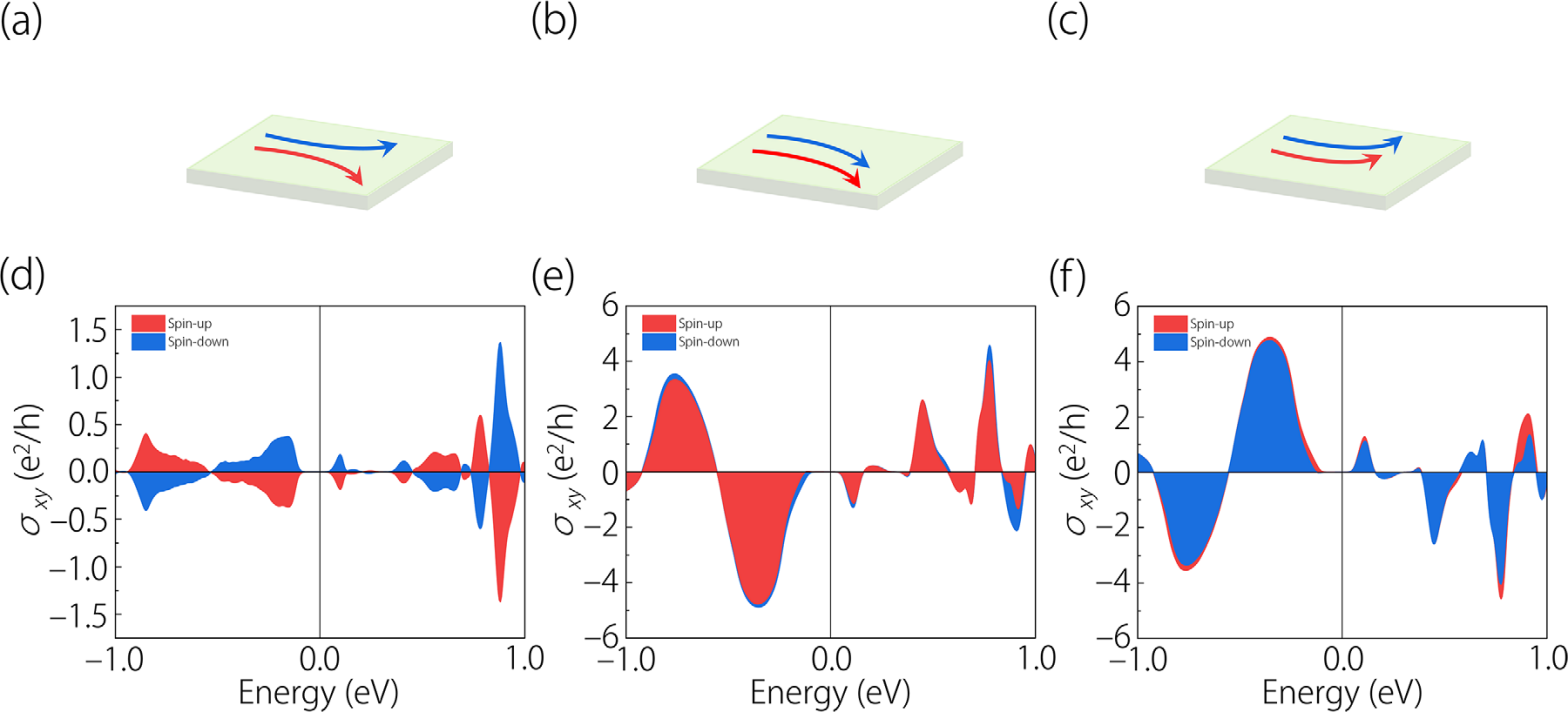
Spin‐resolved charge conductivity of different ferroelectric states. (a–c) The schematic illustration of transport response at 0.01 eV for *F*_A_, *F*_B_, and *F*_C_ states, respectively. (d–f) Corresponding spin‐resolved charge conductivity of *F*_A_, *F*_B_, and *F*_C_ states, respectively.

Based on the Boltzmann transport theory, variations in the band structure under nonequilibrium conditions lead to different carrier distributions, which in turn produce characteristic electrical signals that can be used to distinguish different ferroelectric states. The calculated spin‐resolved charge conductivities are presented in Figure 5. For the *F*_A_ state, the two spin channels exhibit opposite signs of conductivity when an electric field is applied along the *x* direction, resulting in a pure spin current with negligible charge flow (Figure 5a,d). In contrast, the *F*_B_ and *F*_C_ states display anisotropic charge conductivities, leading to finite net charge currents due to the modified spin splitting pattern across the first Brillouin zone. Notably, as shown in Figure 5e,f, not only are the magnitudes of conductivities altered, but their signs are completely reversed, and the spin channels are interchanged. These distinct transport responses thus provide a practical electrical signature for identifying the different ferroelectric configurations. Finally, it should be noted that the spin‐resolved charge transport features are symmetry‐protected. Variations in the electronic relaxation time affect only the magnitude of the conductivity, leaving the qualitative behavior and the sign reversal between states unchanged, as shown in Figure S8.

Before closing this section, we note that the Ti_3_Se_3_Te_2_ bilayer provides a rare multifunctional platform, in which the ferroelectric switching, the reorientation of the easy‐magnetization direction, and the tuning of nonrelativistic spin splitting are intrinsically intertwined and evolve cooperatively. The sliding‐induced ferroelectric states not only determine the system's easy magnetization axes but also reshape the momentum‐dependent spin‐splitting characteristics of AMs, a feature seldom reported in previous studies. This electrically switchable altermagnetic landscape offers a unique and efficient avenue for modulating magnetic anisotropy and controlling spin splitting without relying on external magnetic fields.

## 3 Conclusion

In this work, we establish 2D van der Waals material Ti_3_Se_3_Te_2_ as a unified platform hosting altermagnetism, QAH and QSH states, and a three‐state in‐plane ferroelectricity. The Ti_3_Se_3_Te_2_ monolayer is a dynamically stable ferromagnet with a *T_C_* of 160 K, whose magnetization direction drives a phase transition between a metal and a QAH insulator with a nonzero Chern number. The AA‐stacking bilayer forms an altermagnetic QSH insulator when its magnetization is aligned along the *z*‐axis. In addition to altermagnetism, the AA′‐stacking bilayer hosts a three‐state in‐plane ferroelectricity, going beyond the usual two‐state out‐of‐plane ferroelectricity. Importantly, switching among these three ferroelectric states effectively modulates both the nonrelativistic spin splitting and the easy‐magnetization axis. Ti_3_Se_3_Te_2_ integrates altermagnetism, three‐state in‐plane ferroelectricity, electrically controllable spin splitting, and an easy‐magnetization axis, and rich topological phases including QAH and QSH phases, establishing it as a highly promising class of multifunctional 2D materials.

## 4 Calculation Methods

Based on DFT, the first‐principles calculations were performed using the Vienna ab initio simulation package (VASP) [48, 49]. The exchange‐correlation effects were treated within the generalized gradient approximation (GGA) using the scheme of Perdew‐Burke‐Ernzerhof (PBE) function [50]. The projector‐augmented wave (PAW) was adopted [51]. The plane‐wave cutoff energy of 500 eV and the convergence criterion for the total energy of 10^−6^ eV were utilized in all calculations. The first Brillouin zone was sampled with a Monkhorst‐Pack *k*‐kesh of 9 × 9 × 1 [52]. The correlation effects of the Ti atom 3*d* orbitals were accounted for using the GGA+U method with an effective Hubbard parameter of U_eff_ = 3 eV [53, 54]. A vacuum layer thicker than 15 Å was adopted to avoid artificial interactions between adjacent slabs, and the interlayer van der Waals interaction was treated using the DFT‐D3 method of Grimme with zero‐damping function [55, 56]. The phonon‐dispersion spectrum was calculated using the PHONOPY [57] code to confirm the dynamical stability, and the thermal stability was addressed at 300K through AIMD calculations. The irreducible representations of electronic states from DFT results were calculated using the IRVSP code [58]. The dipole correction was applied along the *z*‐axis to eliminate the spurious interaction between periodic images, and the ferroelectric transition pathway was obtained by the climbing image nudged elastic band (CI‐NEB) method [44, 45]. Based on the DFT band structures, the maximally localized Wannier functions (MLWFs) were constructed with the WANNIER90 package [59, 60, 61], using initial projections from the *s*, *p*, and *d* orbitals of Ti and the *s* and *p* orbitals of Se and Te to ensure good interpolation. The spin‐resolved charge conductivity is calculated using the WANNIER90 package within the Boltzmann transport theory [62], with the electronic temperature and relaxation time set to 100 K and 10 fs. The WannierTools package was utilized to calculate the edge states, WCC, intrinsic AHC, and spin Hall conductivity (SHC) [63, 64, 65].

The intrinsic AHC is given as the first Brillouin zone integral of Berry curvature weighted by the occupation factor of each state,

(2) $$\sigma_{xy}^{A}=-\frac{e^{2}}{\hbar V}\sum_{n,k}\Omega_{xy}^{n}\left(k\right)f_{nk}$$

where *n*, *k*, and *f_nk_* are the band index, wave vector, and Fermi‐Dirac distribution function, respectively [66]. The Berry curvature $\Omega_{xy}^{n}\left(k\right)$ is defined as:

(3) $$\Omega_{xy}^{n}\;\left(k\right)=-\sum_{m\neq n}\frac{2Im\left[\langle \psi_{nk}|{\hat{v}}_{x}|\psi_{mk}\rangle \langle \psi_{mk}|{\hat{v}}_{y}|\psi_{nk}\rangle \right]}{\left(\varepsilon_{nk}-\varepsilon_{mk}\right)}$$

where *n*(*m*), |ψ_*nk*_〉(|ψ_*mk*_〉), ε_*nk*_(ε_*mk*_) and ${\hat{v}}_{x}\left({\hat{v}}_{y}\right)$ are the band index, eigenstates, eigenvalues of the Hamiltonian, and the velocity operator, respectively [66].

The intrinsic SHC is calculated by the Kubo–Greenwood formula [67, 68]:

(4) $$\sigma_{xy}^{S}=h\int_{BZ}\frac{d^{3}k}{{\left(2\pi \right)}^{3}}\sum_{n}f_{nk}\times \sum_{m\neq n}\frac{2Im\left[\langle \psi_{nk}|{\hat{j}}_{x}^{S_{z}}|\psi_{mk}\rangle \langle \psi_{mk}|-e{\hat{v}}_{y}|\psi_{nk}\rangle \right]}{{\left(\varepsilon_{nk}-\varepsilon_{mk}\right)}^{2}-{\left(\hbar \omega +i\eta \right)}^{2}}$$

where BZ is the first Brillouin zone, *f_nk_* is the Fermi distribution function, n and m are the band indexes, ε_*n*_ and ε_*m*_ are the eigenvalues, ${\hat{j}}_{x}^{S_{z}}$ is the spin current operator in the projection of the spin *z* direction (*S_z_*), ${\hat{v}}_{y}$ is the velocity operator, and both the angular frequency ω and the damping factor η are reduced to zero in the scenario of direct current with a clean limit.

To investigate the magnetic properties of the Ti_3_Se_3_Te_2_ monolayer. We performed calculations using the TB2J package, which is based on the Green's function formalism [69]. The input files for TB2J are calculated by using the Atomic Orbital Based Ab‐initio Computation at USTC (ABCUS) package [70, 71]. The ABACUS Pseudopotential Numerical Atomic Orbital Square (APNS) set of optimized norm‐conserving Vanderbilt (ONCV) pseudopotentials and the PBE exchange‐correlation functional are adopted in the calculations [50, 71, 72, 73, 74]. The Kohn‐Sham wave functions were expanded using a linear combination of atomic orbitals (LCAO) corresponding to APNS ONCV [71, 72, 73, 74]. The orbits and corresponding pseudopotentials are available for download from the ABACUS website. The real‐space Heisenberg exchange parameters are given by:

(5) $$J_{ij}=\frac{1}{4\pi }\;\int_{-\infty }^{E_{F}}dEImTr\left[\Delta_{i}G_{ij}^{↑}\left(E\right)\Delta_{j}G_{ji}^{↓}\left(E\right)\right]$$

where, Δ_*i*_ denotes the on‐site exchange splitting and $G_{ij}^{\sigma }\left(E\right)$ represents the spin‐resolved Green's function. The full set of exchange constants {*J_ij_*} defines a classical Heisenberg Hamiltonian:

(6) $$H=-\sum_{i\neq j}J_{ij}S_{i}\cdot S_{j}$$

This Hamiltonian was solved using the VAMPIRE atomistic spin dynamics code [75]. Simulations are performed on five supercells of different sizes, and the system evolution was carried out using a stochastic spin integrator. The temperatures were sampled from 0 K to 400 K, with each temperature equilibrated over 1 × 10^4^ steps. The *T_C_* was identified from the point of maximum slope in the magnetization curve *M*(*T*) for different system sizes, and the final *T_C_* of the system is then determined by finite‐size extrapolation to infinite size.

## Conflicts of Interest

The authors declare no conflict of interest.

## Supporting information



Supporting File: advs75243‐sup‐0001‐SuppMat.docx

## Data Availability

The data that support the findings of this study are available from the corresponding author upon reasonable request.
