# Supplementary material for: Diverse Landscape of Tunable Magnetic, Topological, and Ferroelectric States in 2D Ti3Se3Te2
Source: Adv Sci (Weinh). 2026 Apr 9:e24385. Online ahead of print. doi: 10.1002/advs.202524385 (PMC13335058; doi:10.1002/advs.202524385)
Supplement: Supplementary file 1 — Supporting File: advs75243‐sup‐0001‐SuppMat.docx [file ADVS-9999-e24385-s001.docx]

Supporting Information

**Diverse Landscape of Tunable Magnetic, Topological, and Ferroelectric States in 2D Ti_3_Se_3_Te_2_**

Jiangtao Yu | Jingbo Bai | Yali Yang | Shifeng Qian* | Xiaotian Wang* | Zhuhong Liu*


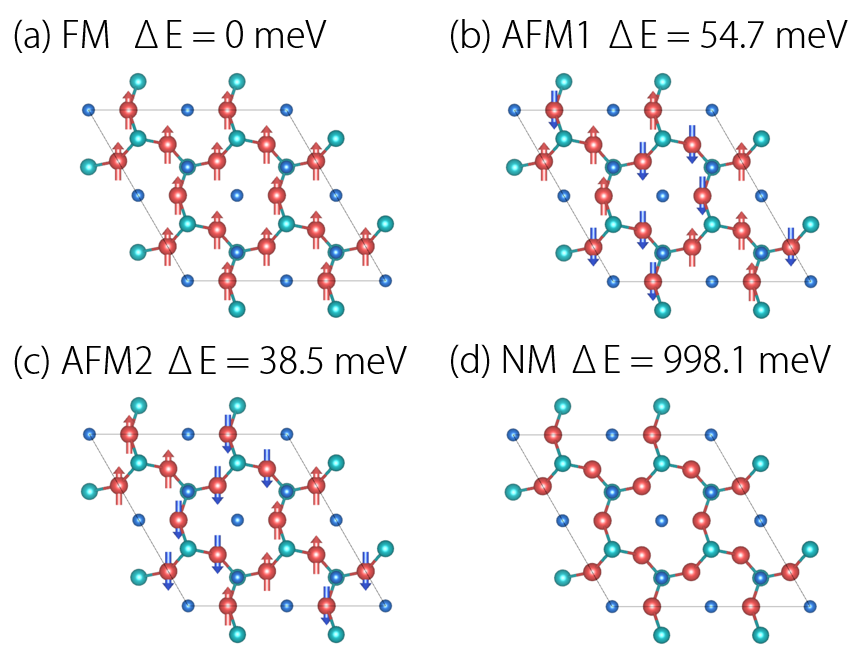


**Figure S1.** Four possible magnetic states of Ti_3_Se_3_Te_2_ monolayer.


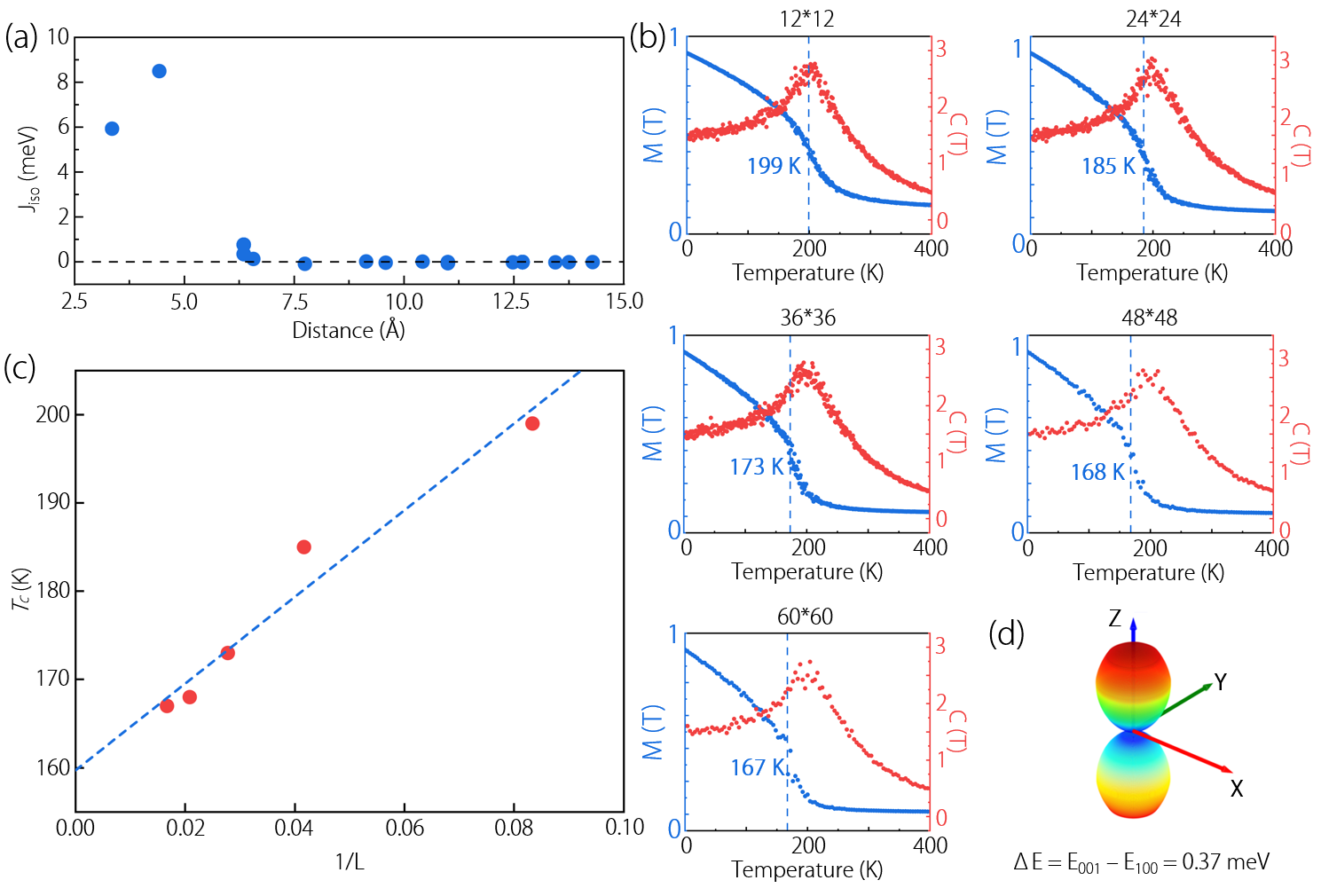


**Figure S2.** (a) Isotropic Heisenberg exchange parameters for Ti_3_Se_3_Te_2_ monolayer. (b) The normalized magnetization and specific heat of Ti_3_Se_3_Te_2_ monolayer as a function of temperature at different system sizes. The Curie temperature *T_C_* was identified from the point of maximum slope in the magnetization curve *M*(*T*). (c) the finite size scaling of the *T_C_*. (d) The magnetic anisotropy energy (MAE) of Ti_3_Se_3_Te_2_ monolayer.

**Table S1.** The formation energy of Ti_3_Se_3_Te_2_ monolayer.

| *E*(Ti_3_Se_3_Te_2_)/*f*.*u*. | *E*(Ti)/*f*.*u*. | *E*(Se)/*f*.*u*. | *E*(Te)/*f*.*u*. | *E_f_*(Ti_3_Se_3_Te_2_)/*f*.*u*. |
| --- | --- | --- | --- | --- |
| –47.207 eV | –7.836 eV | –3.504 eV | –3.144 eV | –6.90 eV |


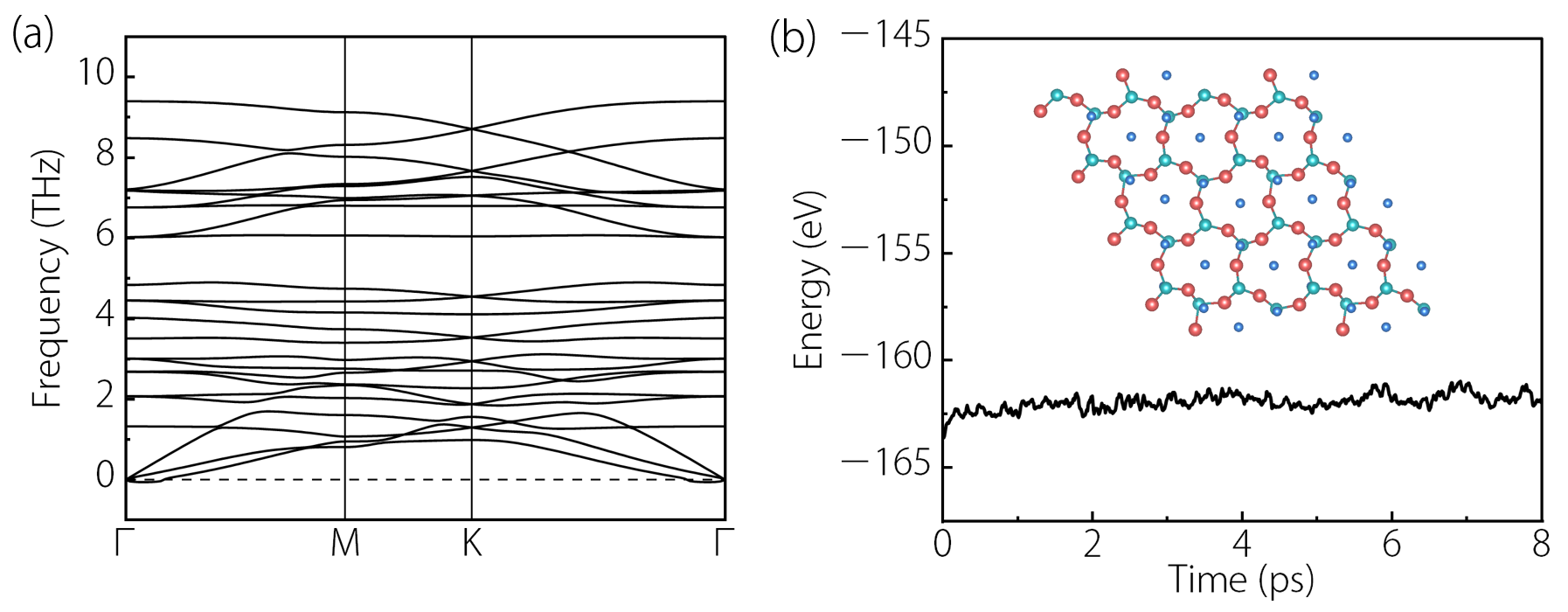


**Figure S3.** (a) The phonon spectrum of Ti_3_Se_3_Te_2_ monolayer. (b) The total energy evolution from *ab initio molecular dynamics* (AIMD) simulations at 300 K, with the inset showing the top-view structure of Ti_3_Se_3_Te_2_ monolayer nanoflake after 8 ps.


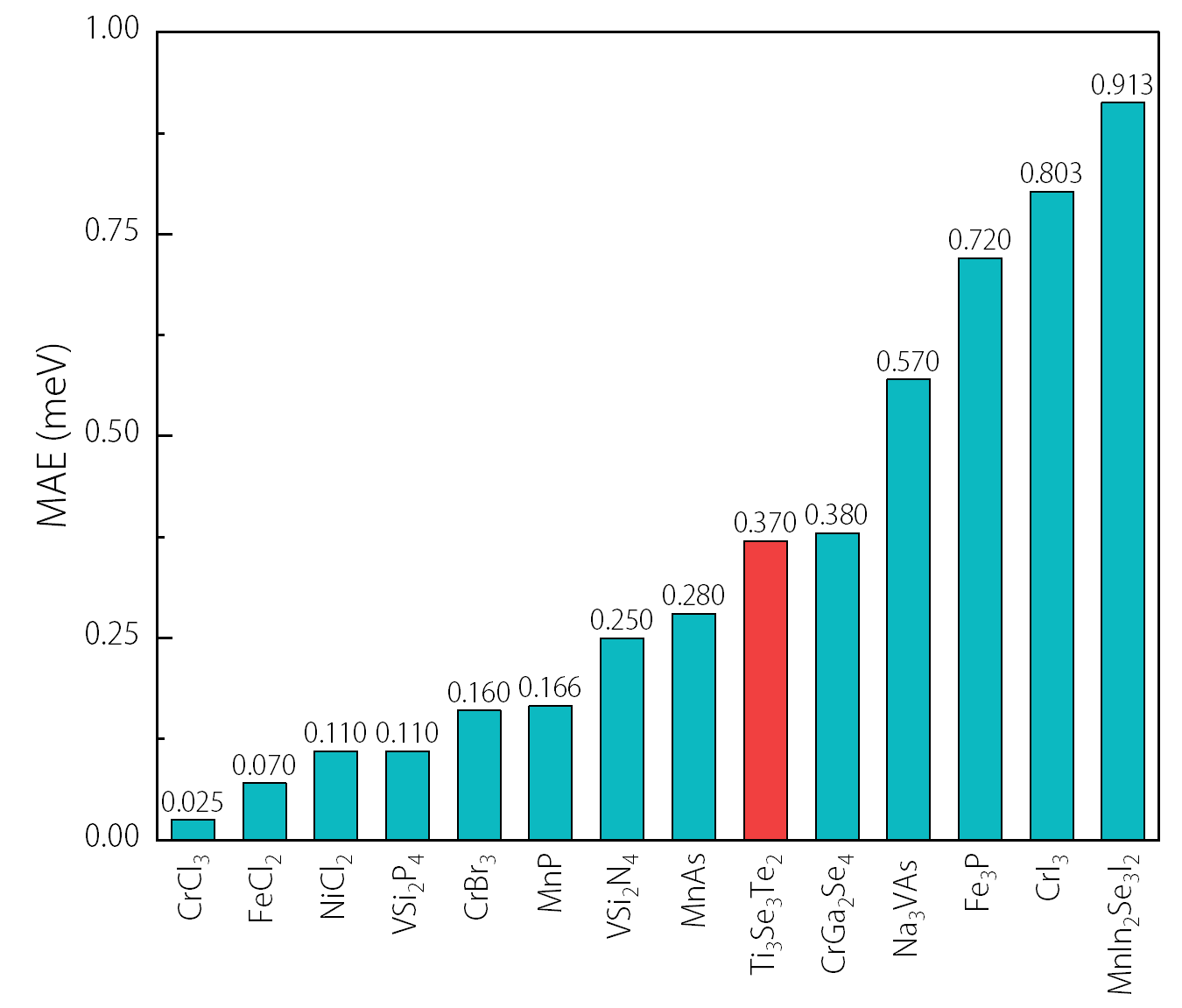


**Figure S4.** Comparison of magnetic anisotropy energy (MAE) per formula unit for representative 2D magnetic materials.


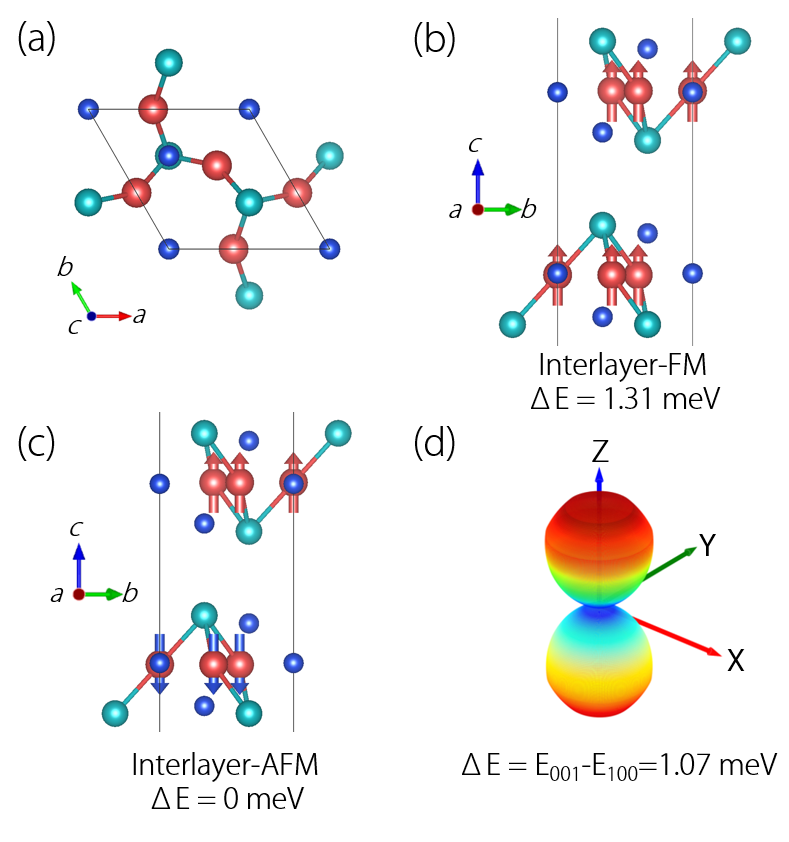


**Figure S5.** (a) The top view of AA stacking Ti_3_Se_3_Te_2_ bilayer. The side views of AA stacking Ti_3_Se_3_Te_2_ bilayer with (b) interlayer-FM and (c) interlayer-AFM. (d) The MAE of AA stacking Ti_3_Se_3_Te_2_ bilayer with interlayer-AFM.

**Table S2.** Relative energies of different magnetic states for the AA-stacked Ti_3_Se_3_Te_2_ bilayer on an h-BN substrate, with the interlayer antiferromagnetic (AFM) state set to 0 meV.

| Magnetic state | Energy (meV) |
| --- | --- |
| interlayer-AFM | 0 |
| interlayer-FM | 13.9 |


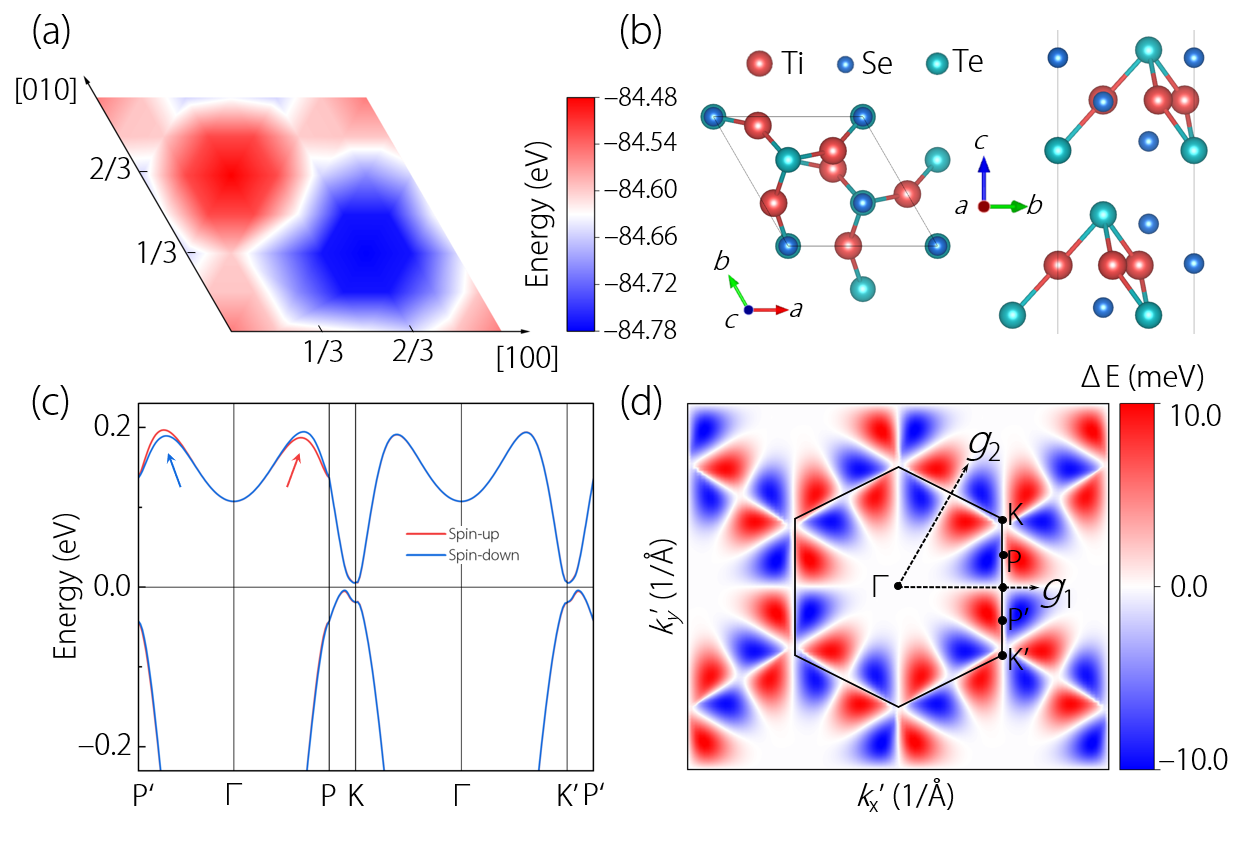


**Figure S6.** (a) The interlayer-AFM energy of AA-stacking for different translations. (b) The top and side views of Ti_3_Se_3_Te_2_ bilayer with the stacking operator of $\hat{P}=\left\{ E\left| \frac{2}{3} \right.,\frac{1}{3} \right\}$. The red, blue and cyan spheres represent Ti, Se, and Te atoms, respectively. Corresponding (c) band structure without SOC and (d) the spin splitting in the first Brillouin zone. The red and blue curves express the spin-up and spin-down channels, respectively. The red and blue areas denote positive and negative spin splitting of conduction band, respectively, while the white indicates regions of spin degeneracy.


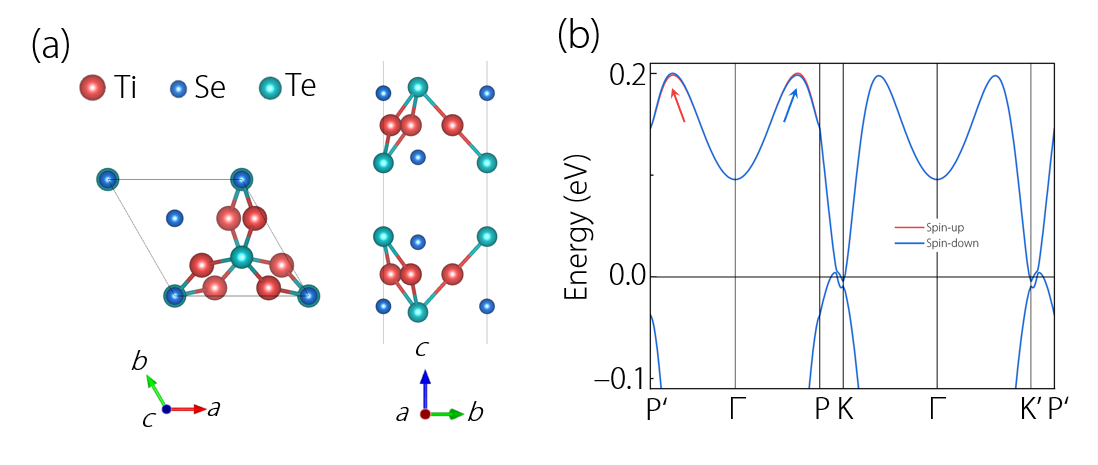


**Figure S7.** (a) The top and side views of AA' stacking Ti_3_Se_3_Te_2_ bilayer. The red, blue and cyan spheres represent Ti, Se, and Te atoms, respectively. Corresponding (b) band structure without SOC. The red and blue curves express the spin-up and spin-down channels, respectively.

**Table S3.** The energy of different magnetic states for AA' stacking of Ti_3_Se_3_Te_2_ bilayer, with the energy of interlayer-AFM normalized to 0 meV.

| Magnetic state | Energy (meV) |
| --- | --- |
| interlayer-AFM | 0 |
| interlayer-FM | 0.852 |

**Table S4.** The energy of different magnetic states for three ferroelectric states, with the energy of interlayer-AFM normalized to 0 meV.

| Ferroelectric state | Magnetic state | Energy (meV) |
| --- | --- | --- |
| *F*_A_ state | interlayer-AFM | 0 |
|  | interlayer-FM | 3.025 |
| *F*_B_ state | interlayer-AFM | 0 |
|  | interlayer-FM | 3.025 |
| *F*_C_ state | interlayer-AFM | 0 |
|  | interlayer-FM | 3.025 |


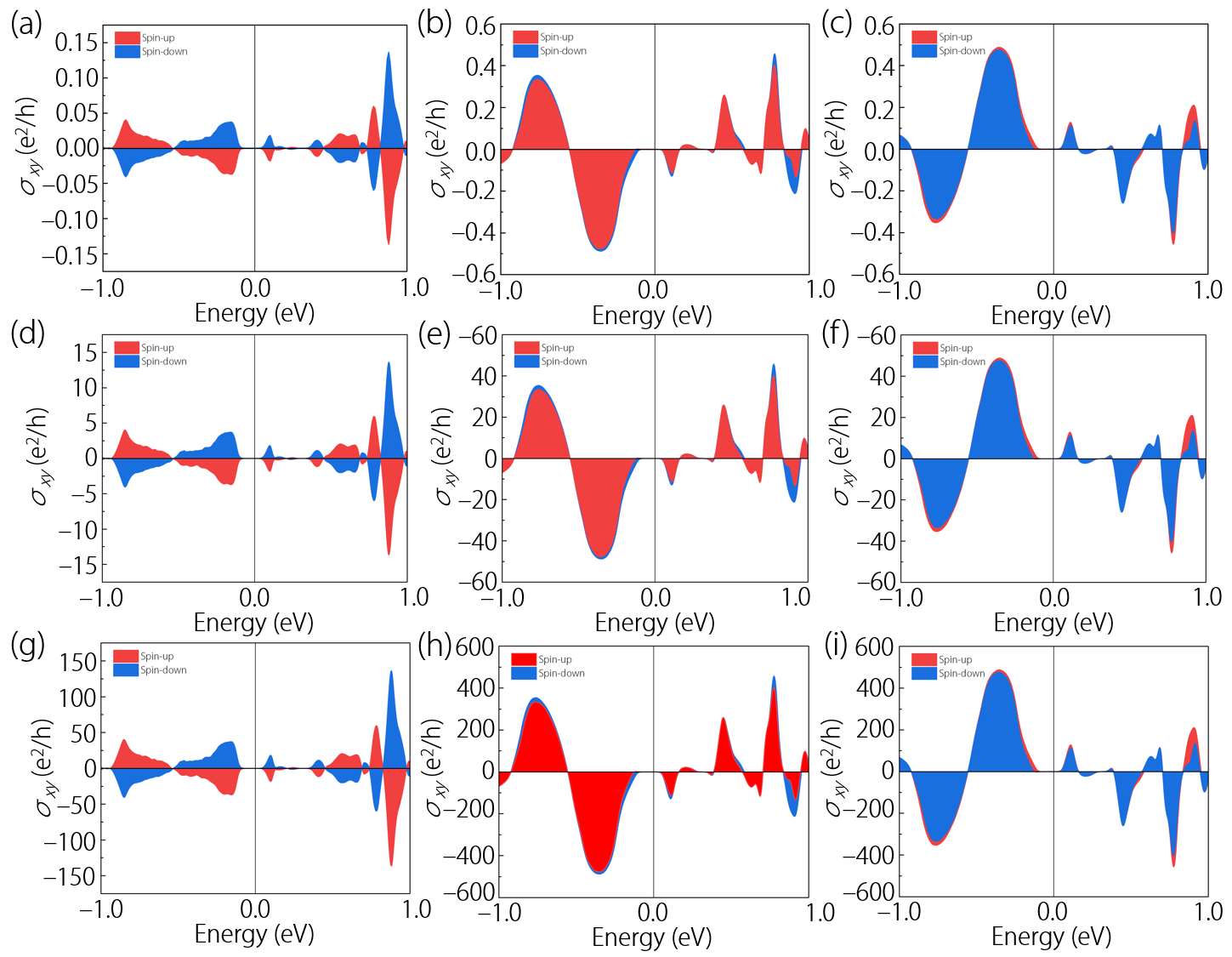


**Figure S8.** Spin-resolved charge conductivity of different ferroelectric states. (a–c) The spin-resolved charge conductivity of *F*_A_, *F*_B_, and *F*_C_ states with the electronic temperature and relaxation time set to 100 K and 1 fs, respectively. (d–f) The spin-resolved charge conductivity of *F*_A_, *F*_B_, and *F*_C_ states with the electronic temperature and relaxation time set to 100 K and 100 fs, respectively. (g–i) The spin-resolved charge conductivity of *F*_A_, *F*_B_, and *F*_C_ states with the electronic temperature and relaxation time set to 100 K and 1 ps, respectively.
